# Human herpesvirus 6 envelope components enriched in lipid rafts: evidence for virion-associated lipid rafts

**DOI:** 10.1186/1743-422X-6-127

**Published:** 2009-08-19

**Authors:** Akiko Kawabata, Huamin Tang, Honglan Huang, Koichi Yamanishi, Yasuko Mori

**Affiliations:** 1 Laboratory of Virology and Vaccinology, Division of Biomedical Research, National Institute of Biomedical Innovation, 7-6-8, Saito-Asagi, Ibaraki, Osaka 567-0085, Japan; 2 Division of Clinical Virology, Kobe University Graduate School of medicine, 7-5-1, Kusunoki-cho, Chuo-ku, Kobe 650-0017, Japan; 3 Department of Pathogenobiology, School of Basic Medical Sciences, Jilin University, Changchun 130021, PR China

## Abstract

In general, enveloped viruses are highly dependent on their lipid envelope for entry into host cells. Here, we demonstrated that during the course of virus maturation, a significant proportion of human herpesvirus 6 (HHV-6) envelope proteins were selectively concentrated in the detergent-resistant glycosphingolipid- and cholesterol-rich membranes (rafts) in HHV-6-infected cells. In addition, the ganglioside GM1, which is known to partition preferentially into lipid rafts, was detected in purified virions, along with viral envelope glycoproteins, gH, gL, gB, gQ1, gQ2 and gO indicating that at least one raft component was included in the viral particle during the assembly process.

## Introduction

Glycolipid-enriched microdomains (GEM) are organized areas on the cell surface enriched in cholesterol, sphingolipids, and glycosylphosphatidylinositol (GPI)-anchored proteins. These areas have been described as "rafts" that serve as moving platforms on the cell surface [1]. These domains exist in a relatively ordered state, which confers resistance to Triton X-100 detergent treatment at 4°C [2].

The infection of host cells by enveloped viruses relies on the fusion of the viral envelope with either the endosomal or plasma membrane of the cell [3]. Therefore, the protein and lipid compositions of both the viral envelope and host cell membrane play crucial roles in virus infection. For all enveloped viruses, the envelope is derived from the host cell during the process of virus budding. Many viruses are known to utilize lipid rafts during budding. Lipid rafts of the plasma membrane function as a natural meeting point for the transmembrane and core components of a phylogenetically diverse collection of enveloped viruses [4]. The rafts are implicated as the areas of the plasma membrane where human immunodeficiency virus type 1 (HIV-1) assembly and budding occur in infected cells [5,6]. In the case of influenza, budding takes place at the apical plasma membrane and is heavily dependent on the presence of lipid microdomains or rafts [7-9]. Measles virus (MV) has also been suggested to use raft membrane in its assembly and budding processes [10,11]. The integrity and organization of cholesterol rich membrane lipid rafts has been suggested to be critical for ordered assembly and release of infectious Newcastle disease virus particles [12].

Human herpesvirus 6 (HHV-6) is a beta herpesvirus and a human pathogen of emerging clinical significance. HHV-6 was first isolated from the peripheral blood lymphocytes of patients with lymphoproliferative disorders and AIDS [13]. HHV-6 isolates can be categorized into two variants, A (HHV-6A) and B (HHV-6B); HHV-6B is the causative agent of exanthem subitum [14]. Recently we have shown that HHV-6 virion buds into TGN derived membrane which has characteristics of late endosome [15].

Here we report that upon membrane fractionation, HHV-6 envelope glycoproteins, glycoproteins H, L, Q1, Q2, O and B (gH, gL, gQ1, gQ2, gO and gB) are present in the detergent-resistant, GM1-rich fractions, confirming their association with lipid rafts. In particular, the mature forms of gQ1, gQ2 and gO, which are expressed only in mature virions, were localized to the detergent-resistant lipid rafts. In addition, HHV-6 virions incorporated the lipid-raft-specific ganglioside, GM1, indicating that HHV-6 virions may assemble through rafts.

## Methods

### Cells and viruses

T-cell lines (HSB-2 cells) were cultured in RPMI 1640 with 8% fetal bovine serum (FBS). HHV-6A strains GS were propagated in HSB-2, and the titers of the viruses were estimated using HSB-2 cells. HHV-6 cell-free virus was prepared as described previously[16]. When HHV-6-infected HSB-2cells showed evidence of more than 80% infection by immunofluorescence assay (IFA), the cells were lysed by freezing and thawing twice, and spun at 1,500 × g for 10 min. The supernatant was used as cell-free virus. Nycodenz gradient-purified virions were obtained as follows. HSB-2 cells were infected with HHV-6, and at 3–4 days postinfection (pi) the infected cells were combined with newly prepared cells for cell-cell spread of HHV-6. At 3–4 days later, the cells were spun at 1,500 × g for 15 min at 4°C. The supernatant from the cells was used for purification of virus particles. The viruses in the supernatant were precipitated with polyethylene glycol (PEG, molecular weight 20,000, 10%) in the presence of NaCl. The viruses were re-suspended, layered over a gradient of 15–60% nycodenz (Sigma), and centrifuged for 1 h at 27,000 rpm in an SW40Ti rotor (Hitachi). The fractions were collected from the bottom. The fractions containing virions were examined by analysis of viral DNA with PCR using primer pair, AgB2232F (5'-acacctagtgttaaggatgttg) and AgBR (5'-tcacgcttcttctacatttac), which could amplify HHV-6A glycoprotein B gene.

### Antibodies (Abs)

The monoclonal antibodies (MAbs) against HHV-6A, anti-gQ1 (AgQ1-119), anti-gQ2 (AgQ2-182), anti-gL (AgL-3) and anti-gO (AgO-N-1), and the mouse antiserum specific for HHV-6A gH were described previously[17]. The rabbit antiserum specific for HHV-6 gB was described previously [15,18]. Anti-CD59 mouse MAb (AbD serotec), anti-Linker for activation of T cells (LAT) mouse MAb (upstate biotechnology), anti-human transferrin receptor (TfR) mouse MAb (Zymed laboratories), anti-CD46 mouse MAb (Immunotech) and anti-CD3zeta mouse MAb (Santa Cruz) were purchased. Cholera toxin B subunit, type Inaba 567B, peroxidase conjugate was obtained from Calbiochem.

### Immunoblotting

The lysed proteins were resolved by SDS-PAGE and electrotransferred onto a polyvinylidene difluoride (PVDF) membrane for immunoblotting. After being blocked, the membranes were incubated for 1 h with blocking buffer (10 mM Tris-HCl [pH 7.2], 0.15 M NaCl, 5% skim milk, 0.75% Tween 20) containing the MAbs or antisera. The reactive bands were visualized using a horseradish peroxidase-linked secondary conjugate and enhanced chemiluminescence detection reagents (GE Healthcare).

### Immunofluorescence assay (IFA)

The IFA was performed as described previously [17].

### Isolation of raft fraction

Raft fractions were prepared as described previously [19,20]. Cells (1 × 10^8^) were washed in PBS and then lysed with 1 mM MES-buffered saline (25 mM MES, pH 6.5, and 150 mM NaCl) containing 1% Triton X-100, 5 mM sodium orthovanadate, and 5 mM EDTA. The lysate was homogenized with 20 strokes of a Dounce homogenizer, and gently mixed with an equal volume of 80% sucrose (w/v) in MES-buffered saline. The sample was then overlaid with 6.5 ml of 30% sucrose and 3.5 ml of 5% sucrose in MES-buffered saline and spun at 200,000 × g at 4°C for 16 h. Following the centrifugation, the fractions were collected from the top of gradient. The fractions were analyzed on a Western blot.

## Results

### Association of HHV-6 proteins with rafts in infected cells

Raft membranes were isolated from HHV-6A-infected T cells (HSB-2) and mock-infected HSB-2 cells using a flotation assay, based on their resistance to solubilization by TX-100 at 4°C and buoyancy at low-density in fractions of a bottom-loaded discontinuous sucrose gradient, with steps of 5, 30, and 40% sucrose.

**Figure 1:**
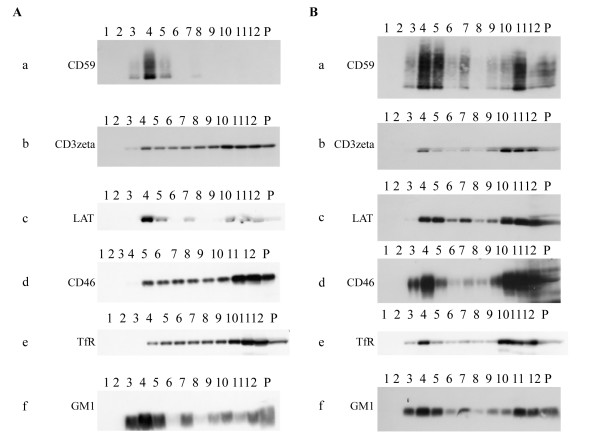
**Isolation of raft membranes from HSB-2 cells; the detection of cellular proteins**. (A) Mock-infected HSB-2 cells. Bottom-loaded sucrose step gradients (fraction 1 represents the top of the gradient) were analyzed by immunoblotting. Immunoblots of proteins from each fraction (equal volume loaded) were labeled with anti-CD59 (a), -CD3zeta (b), -LAT (c), -CD46 (d) or -TfR (e) antibody for cellular proteins. GM1(f), which migrated with the dye front, was detected by reaction with HRP-coupled cholera toxin. (B) HHV-6A, strain GS-infected HSB-2 cells. HSB-2 cells were infected with GS, at 4 days later, the cells were combined with newly prepared cells, and the step was repeated. When HHV-6 infected HSB-2cells showed evidence of more than 80% infection by immunofluorescence assay (IFA), the cells were harvested for the isolation of raft fractions. The population of infection was examined by the expression of late proteins (gQ1, gQ2, gB and gL). P indicates pellet. The experiment was done three times independently, and one of three experiments was shown here. All of these blots came from the same experiment, but the exposure time of each blot was not identical.

As shown in Fig. 1A, in mock-infected cells, the GPI-anchored CD59 protein (a) and linker for activation of T cells (LAT) protein (c) were mainly detected in detergent insoluble fractions which indicate lipid rafts with strongest signal in fraction 4, while transferrin receptor (TfR) protein (e) which is a nonraft marker, CD3zeta protein (b) and CD46 protein (d) were distributed broadly with stronger signals in fractions 10–12 which are detergent soluble fractions. The binding of the cholera toxin β subunit (CTx), which specifically detects the raft-associated glycosphingolipid GM1 (f), was mostly partitioned into fractions 3–5 with strongest signal with fraction 4. Therefore, the rafts were mostly recovered in fractions 3–5 in mock-infected HSB-2 cells. Fig. 1B shows that in HHV-6A-infected HSB-2 cells, CD59 protein (a), which is concentrated in lipid rafts was mostly migrated to fractions 4–5 and 11 with strongest signals in fractions 4–5. LAT protein (c), which is concentrated in lipid rafts, was also detected in fractions 4–5, 10,11 and 12 in infected cells. Similarly, the raft-associated glycosphingolipid GM1(f), was partitioned into fractions 3–5, 11, 12 and pellet with the strongest signals in fractions 4. Therefore, the rafts were mostly recovered in fractions 3–5, especially in fraction 4 of HHV-6 infected cells, which we referred to as the raft fractions. Insoluble cytoskeleton components and nuclear remnants were recovered in the pellet at the bottom of the tube. Interestingly, CD46 protein (d), which is a cellular receptor of HHV-6, also migrated to fraction 3–5 with strongest signal in fraction 4 after HHV-6 infection. CD3zeta (b) and TfR (e) proteins were also detected in fraction 4 with stronger signal in HHV-6-infected cells. They may be migrated into raft fractions after infection. These results showed that non-raft proteins could be migrated into raft fractions after infection, suggesting that the cellular machinery may be modified by HHV-6 infection.

**Figure 2:**
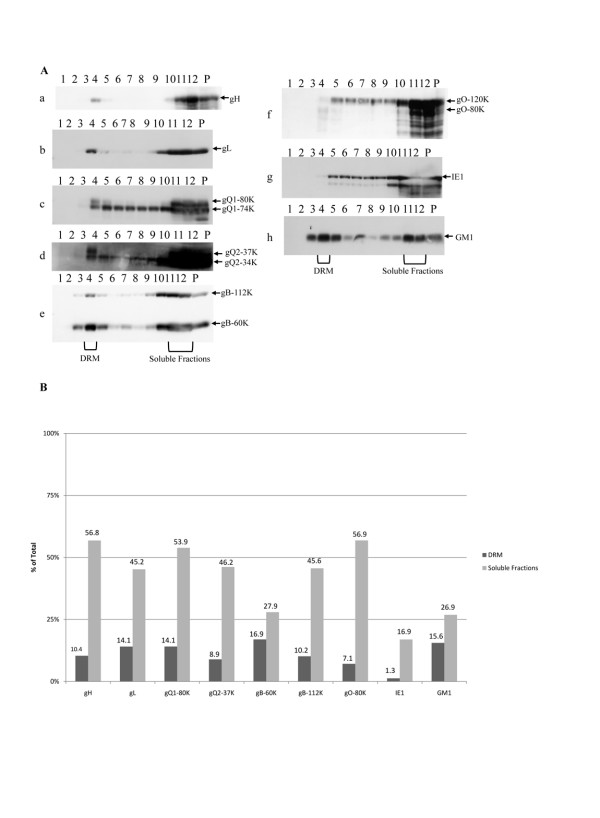
**Isolation of raft membranes from HSB-2 cells infected with HHV-6A; the detection of viral proteins**. Bottom-loaded sucrose step gradients (fraction 1 represents the top of the gradient) were analyzed by immunoblotting. (A) Immunoblots of proteins from each fraction (equal volume loaded) were labeled with anti-gH (a), gL (b), gQ1-80K(c), gQ1-74K(c), gQ2-37K(d), gQ2-34K(d), gB-112K (e), gB-60K(e), gO-120K(f), gO-80K (f), or IE1(g) antibody for HHV-6A proteins. GM1 (h), which migrated with the dye front, was detected by reaction with HRP-coupled cholera toxin. Same photo used in Fig. 1B (f) was used for GM1. The positions of the viral proteins are indicated on the right of the figure. P indicates pellet. The sample loaded here was same one used in Fig. 1B. The experiment was done three times independently, and one of three experiments was shown here. All of these blots came from the same experiment, but the exposure time of each blot was not identical. (B) The blots were quantitated by densitometry analysis by KODAK MI software and the amounts in DRM or soluble fractions were determined as a percentage of the total of all the blots. Number indicates each percentage. DRM; detergent resistant membrane.

Next, we examined the raft association of viral glycoproteins in HHV-6A-infected HSB-2 cells. As shown in Fig. 2A, a proportion of the HHV-6 envelope glycoproteins, glycoprotein H (a), L(b), Q1(c), Q2(d), B(e) and O(f) (gH, gL, gQ1, gQ2, gB and gO) colocalized with the raft fractions (Fig. 2A). The distribution between DRM and soluble fractions was quantitated by KODAK MI software (Fig. 2B). Interestingly, although gQ1-74K, gQ2-34K and gO-120K were broadly distributed in fractions, the mature forms 80-kDa gQ1 (gQ-80K), 37-kDa gQ2 (gQ2-37K) and 80-kDa gO (gO-80K) proteins that contain complex type N-linked oligosaccharide and are expressed in mature virions[17,21,22] were distributed in fraction 4 (14.1%, 8.9% and 7.1% respectively) in addition to fractions 11–12 (53.9%, 46.2% and 56.9 respectively), indicating that the mature forms of gQ1, gQ2 and gO co-localized with the lipid rafts. In contrast, almost of the nonstructural protein, the immediate early 1 (IE1) protein (g), which is mainly expressed in the nucleus and cytoplasm, was recovered from the soluble fractions (fractions 5–12) and the pellet, but it was rarely recovered from fraction 4 (1.3%).

### Raft membranes are included in the HHV-6 envelope

**Figure 3:**
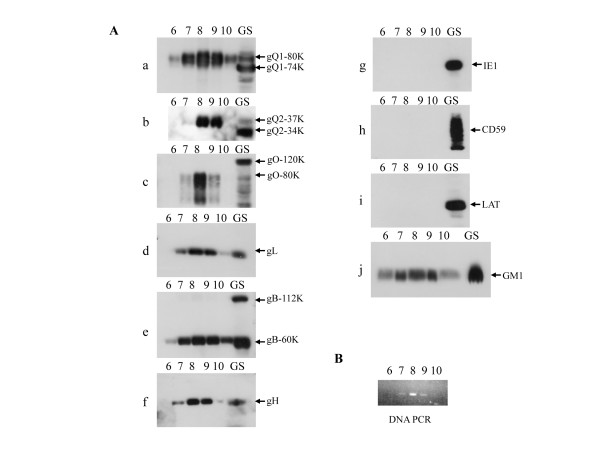
**The glycosphingolipid GM1 was detected in purified mature virions**. Virions were purified by a two-step nycodenz gradient method. (A) Each fraction was analyzed by immunoblotting. Immunoblots of the proteins in each fraction (equal volume loaded) were labeled with anti-gQ1(a), -gQ2(b), -gO(c), -gL(d), -gB(e), -gH (f) or -IE1(g) MAb for viral protein, and anti-CD59 (h) or -LAT (i) MAb. GM1 (j) was detected by reaction with HRP-coupled cholera toxin. The numbers above each column represent the fraction from the bottom of the gradient. The fractions were collected from the bottom. GS indicates the extracts from GS-infected HSB-2 cells. GM1, but not CD59 or LAT was detected in the virion fractions. (B) PCR was performed for analysis of viral DNA in the fractions. The fractions 7–9 contained viral DNA.

HHV-6 obtains its lipid envelope from the host cell membrane during the maturation process of the virions. We next investigated whether raft-associated HHV-6 proteins contributed to HHV-6 envelope maturation and were incorporated into the viral envelope. Viruses released from HHV-6A-infected cells were purified twice by nycodenz gradient. The fractions were collected from the bottom. The fractions containing virions were determined by analysis of viral DNA with PCR (Fig. 3B), and the results suggested that the fraction 8 contained most abundant virions

As shown in Fig. 3A, the ganglioside GM1 (j) was detected in fractions containing virions, similar to the other HHV-6 envelope glycoproteins (Fig. 3A-a, b, c, d, e and 3A-f) indicating that the HHV-6 envelope contains lipid rafts. However, CD59 (h) and LAT (i), proteins that were detected in the raft fractions of HHV-6-infected cells, were not recovered from the virion fractions as well as IE1(g) which is not a viral structural protein, indicating that these host proteins expressed in lipid rafts are not incorporated into viral particles.

## Discussion

In this study, we found that raft membranes contain a proportion of viral envelope proteins at late phase in HHV-6 infected cells.

Previously, we reported that HHV-6 gQ1 and gQ2 each exist in two forms, gQ1-74K, gQ2-34K and gQ1-80K, gQ2-37K respectively, and that only gQ1-80K and gQ2-37K, which contain complex type N-linked oligosaccharides, are incorporated into viral particles [17]. Furthermore, we reported that HHV-6 gO also exists in two forms, gO-120K and gO-80K, and gO-80K contains complex type N-linked oligosaccharides, are incorporated into viral particles[22]. Here we observed that a subpopulation of HHV-6 envelope proteins, gH, gL, and gB, and interestingly, a subpopulation of the mature proteins gQ1-80K, gQ2-37K and gO-80K are associated with rafts, but the nonstructural protein, IE1 remains excluded from raft membrane, indicating that as the HHV-6 envelope glycoproteins mature through the endoplasmic reticulum (ER) and Golgi, the raft association of HHV-6 glycoproteins may occur during the maturation step in post Golgi compartment. Since lipid rafts occur in the Golgi complex[23], such a glycoprotein concentrating function of lipid rafts may also be significant for efficient beta herpesvirus budding and particle formation as has been hypothesized in alpha herpesviruses[24,25]. The budding of the HHV-6 has been reported to be preceded by the assembly of viral components at special sites that are TGN-derived vesicles [15]. Therefore, we speculate that lipid raft microdomains may provide a cellular location for HHV-6 assembly in infected cells.

Interestingly, in mock-infected HSB-2 cells, CD59 and LAT proteins, which are concentrated in lipid rafts were tightly migrated to fraction 4, while in HHV-6-infected cells, they were migrated to fractions 4, 5, 10, 11 and 12. Because cellular machinery is possibly modified at the late phase of infection and the structure of cellular membrane appears to become loose, CD59 and LAT may have been also detected in fractions 10, 11 and 12 in addition to fractions 4 and 5.

We show here that HHV-6 virions incorporated GM1 as well as virus envelope proteins, but not CD59 and LAT, indicating that the raft membrane was incorporated into viral particles. HIV-1 particles carry the lipid-raft-specific ganglioside GM1 and a number of cellular GPI-anchored proteins, such as CD59, on their surface [6]. This incorporation of particular cell membrane constituents is likely to be a direct consequence of the preferential budding of HIV-1 through the so-called raft microdomains of the plasma membrane [6].

Herpes simplex virus (HSV) tegument protein, virion host shut-off protein (vhs) appears to be associated with lipid rafts, and this raft population is enriched in a cytoplasmic membrane fraction, which contains assembling and mature HSV particles, and the raft association of vhs is speculated to correlate with the assembly of vhs into tegument [25]. HSV-2 UL56p is also reported to associate with rafts [26]. By using detergent solubilization experiments, HSV gB, but not gC, gD or gH has been shown to localize to raft fractions during virus entry [27]. Pseudorabies virus (PRV) gB has been show to be a strong, detergent-resistant raft association whereas gC and gD not to be strong lipid raft association in PRV-infected cells [28]. Our results suggest that HHV-6 mature virions may bud through lipid rafts in TGN-derived vesicles, thus incorporating host-cell cholesterol and sphingolipids.

## Competing interests

The authors declare that they have no competing interests.

## Authors' contributions

AK and YM carried out all analyses, AK, HH, HT and YM carried out the research, KY analyzed the study, AK and YM participated in written of the manuscript. All authors have read and approved the final manuscript.
